# Perspectives and experiences of community health workers in Brazilian primary care centers using m-health tools in home visits with community members

**DOI:** 10.1186/s12960-017-0245-9

**Published:** 2017-09-29

**Authors:** Julia Schoen, John William Mallett, Rebecca Grossman-Kahn, Alexandra Brentani, Elizabeth Kaselitz, Michele Heisler

**Affiliations:** 1 University of Michigan Medical School, 1301 Catherine St, Ann Arbor, MI 48109 United States of America; 2 Department of Pediatrics, University of São Paulo Medical School, Av. Dr. Arnaldo, 455 - Cerqueira César, São Paulo, SP 01246-903 Brazil; 3 Center for Clinical Management Research, Ann Arbor Veterans’ Affairs (VA) Healthcare System, 2800 Plymouth Rd, Ann Arbor, MI 48109 United States of America; 4 Department of Internal Medicine, University of Michigan Medical School, 1301 Catherine St, Ann Arbor, MI United States of America; 5 Department of Health Behavior and Health Education, School of Public Health, University of Michigan, 1415 Washington Heights, Ann Arbor, MI 48109 United States of America

**Keywords:** Community health workers, Brazil, Primary care, Community health, m-health, Qualitative research

## Abstract

**Background:**

Mobile health (m-health) tools are a promising strategy to facilitate the work of community health workers (CHWs) in low- and middle-income countries (LMICs). Despite their potential value, little is known about CHWs’ experiences working with m-health tools in their outreach activities with community members.

**Methods:**

To understand the benefits of and barriers to using m-health tools for CHWs, we conducted semi-structured interviews with 57 CHWs employed in six primary care centers in São Paulo, Brazil. All CHWs had experience using a cell phone application called Geohealth for collecting health and demographic data of community members. We assessed their experiences using Geohealth and recommendations for improvements.

**Results:**

CHWs described key benefits of using Geohealth as helping them save time with bureaucratic paperwork, organizing the data that they needed to collect, and by replacing sheaves of paper, reducing the weight that they carried in the field. However, there were many technical and social barriers to the successful adoption of the m-health tool. Key among these were poor quality hardware, faulty software programs, and negative community member perceptions of the m-health program. The CHWs provided valuable input as to how Geohealth could be improved to fit their needs.

**Conclusion:**

m-health tools have the potential to facilitate the work of CHWs in LMICs. However, such tools must be designed and implemented thoughtfully. Technical barriers related to both hardware and software must be anticipated and addressed to maximize their efficiency and successful adoption. CHW input on the design of the tool should be sought to maximize its utility and minimize barriers to use.

## Background

A key strategy for improving health in low- and middle-income countries (LMICs) is to strengthen primary care health systems. One promising approach to improve community-based primary care is to incorporate community health workers (CHWs) into health care teams. CHWs can serve as liaisons between communities and health systems and augment disease prevention and management efforts in the communities they serve [1, 2]. CHW outreach has been effective across a variety of illnesses and in a number of different countries [3–8].

m-health strategies aim to help CHWs address gaps in healthcare access in low-resource settings [9, 10] and improve the efficiency and quality of care provided. A number of m-health strategies for CHWs have been developed in recent years [11]. These tools often are designed to help CHWs collect health data, receive reminders in the field, facilitate community member education, improve communication [12], and facilitate emergency referrals [11]. A small but growing body of research has shown that m-health tools provide value to CHWs and patient care [11, 13–15]. However, few efforts have been made to incorporate such tools into standard practice [11, 16]. Schuchman et al. (2014) refers to this as m-health ‘pilotitis’ or a proliferation of m-health pilot studies that are not successfully implemented or tested to scale. Moreover, there is little information on the experiences and perspectives of CHWs themselves using m-health tools. This type of qualitative research may help explain why m-health interventions often do not progress past the pilot study phase.

The Family Health System (FHS) in Brazil provides an excellent example of real-life implementation of m-health tools into the routine work of CHWs. In the FHS, community primary care clinics employ teams of health professionals responsible for community members in a particular neighborhood. Each team consists of a physician, a nurse, two nurse assistants, and six full-time, salaried CHWs, recruited from the neighborhoods they serve, who make monthly home visits with community members receiving care at the primary care center. One aspect of the CHW job is to collect information about community members’ social and health conditions and needs. This information is relayed to the clinic and in turn compiled into a governmental statistical database called SIAB. In some health centers, efforts have been made to develop and test m-health information systems for CHWs [17].

One example is an initiative in the Western Region of the city of São Paulo developed and implemented between 2009 and 2015 in six primary care centers administered by the University of São Paulo’s Medical School via their non-profit organization Fundação Faculdade de Medicina [18, 19]. The system, called Geohealth, used both a mobile application and computer program to increase the efficiency of data collection during home visits and the transmission of information to the SIAB database [20]. CHWs entered data either using a mobile phone application or computer application, and the data were sent to a secure remote server. Nurses, physicians, and the CHWs could use the computer program to map demographic and health information from the Geohealth database. The mobile application was designed to improve CHWs’ workflow by eliminating the shortcomings of collecting data on paper, including lack of standardization, loss of paper documents, and limited data security. In addition to the forms required by the municipality, Geohealth included extra fields designed by the research group, such as primary care enrollment refusals, deaths, and geographical coordinates to be used as mapping references.

To date, little is known about how the CHWs used the Geohealth mobile application, the barriers and facilitators to its use, and CHW’s recommendations for ways to improve the application. To fill this gap in knowledge, we conducted semi-structured interviews among CHWs working in six primary care centers in the Western Region of the city of São Paulo to evaluate three key research questions:

1. What were the experiences of the CHWs with the Geohealth mobile application and computer program?
2. What were the main strengths and weaknesses as well as barriers and facilitators to effective use?
3. How can the system be improved to facilitate CHWs’ daily work?

Insights gained through this study will help inform future efforts to improve the design and implementation of m-health tools for CHWs in Brazil and other LMICs. Moreover, although the Geohealth application is not currently being used due to a restructuring of the FHS in the Western Region, the municipality of São Paulo is creating a municipal-level electronic system, and many features of the Geohealth system will be used in this system.

## Methods

This study explored the experiences of Brazilian CHWs using the Geohealth mobile application and computer program through qualitative semi-structured interviews. A qualitative approach was used to understand the nuanced and complex experiences of CHWs using this m-health tool in their daily work. This method was selected to provide a complete picture of how Geohealth fits into the CHW’s daily tasks without excluding factors potentially affecting CHW viewpoints. While using a qualitative approach ultimately limited our capacity to quantify data, it also allowed for a better appreciation of the real-world complexity of situations m-health users experience. The Institutional Review Boards for the study sites approved this research.

### Setting and participants

The six study health centers serve low-income neighborhoods of São Paulo. Together, the study health centers employed 31 healthcare teams and served 95 903 community members.

We conducted one-on-one interviews at five health centers and a focus group with 10 CHWs at a sixth center. The interviews and focus group explored the CHWs’ experiences and their use of Geohealth. The questions were designed to understand how Geohealth influenced the efficiency and effectiveness of the CHWs, as well as their satisfaction with their work. The interviews also covered CHWs’ satisfaction with Geohealth’s user-design interface. Three members of the research team (JHS, RGK, and JWM) conducted all interviews in Portuguese. Permission was obtained from every health center’s manager before arriving on site, and verbal informed consent was obtained from each participant. Table 1 shows characteristics of the interviewed CHWs.

**Table 1:** Participant baseline characteristics (*N* = 57)

Age, mean (years):	41
Female (%):	91
Mean time as CHW (years):	6
Mean time using Geohealth (years):	2

### Data analysis

Interview notes were taken by hand and subsequently typed and translated into English. We used standard methods of qualitative research to analyze the transcripts using codes and sub-codes. A preliminary codebook was developed using an iterative process until all team members reached agreement [21, 22]. Each transcript was coded by three researchers to resolve discrepancies and refine the codebook. The most prevalent codes were analyzed to develop the key themes. All team members reviewed a completed transcript with the recurring themes and illustrative quotations to ensure validity.

## Results

### CHW experiences with the Geohealth mobile application and computer program

This study initially focused on the CHWs’ experiences using the mobile application and recommendations for improving it. However, CHWs used the term “Geohealth” interchangeably to refer to both the computer program and the mobile application, viewing them as separate components of one Geohealth system. The research’s scope was expanded to characterize how the CHWs used the system and to clarify how the mobile application and computer program fit into their workflow. Table 2 shows illustrative quotes from interviewed CHWs on their experiences using the mobile application.

**Table 2:** Experiences of the CHWs with the Geohealth mobile application

Many ways of using Geohealth	I’ve been using Geohealth for 3 years. I use it on home visits to register families, to look up things about my area like how many kids I have in my area. I write the summaries in the homes by hand, and then I transfer it to the Geohealth system using a cell phone or computer. Usually computer because my Geohealth phone broke. I don’t use [Geohealth] in home visits. For example, let’s say today I did 10 visits. Tomorrow I do 10 more. At the end of tomorrow, I’ll sit down and put all 20 visits into the computer. I don’t like typing on the phone. It is better at a computer. Sometimes I’m in the middle of a visit, and then another community member comes along and I have to answer a quick question for them—but there is no way to do these two visits simultaneously on Geohealth. Sometimes it takes 15 min just to turn on, or to find a SUS [identification] number in the system—that’s the time my entire visit should take. I use the computer 90% of the time for Geohealth.
Organization	I use Geohealth to register families, and to update information about families. If there is a new baby or new family member, or death. Families are always changing… Each month a new family arrives or leaves.
Accountability	I still use paper to collect signatures at every house I visit to prove that I went to the house. You have situations where a community member goes straight to the health center asking for an appointment and says that a CHW never visited. I can show them that they signed on such and such day proving that I did go there. This helps avoid a lot of confusion. It is a strategy community members have: to say we didn’t go to their house to try to get seen sooner. We need to account for every house we visit.

#### Many ways of using Geohealth

The CWHs ranged greatly in the extent to which they used Geohealth in their everyday work. They developed ways to combine Geohealth technology with paper records. CHWs used either the computer program or the mobile application to enter information into the Geohealth database. Only a few used the mobile application consistently during home visits. The majority took notes on paper during the home visit and then entered their notes in the Geohealth application at the health center.

#### Organization

The CHWs used Geohealth to organize their data. For example, they used Geohealth to register new families and to generate monthly counts of community members in their area with certain characteristics such as diabetes.

#### Accountability

Geohealth provided accountability to CHWs. The CHWs reported that they needed a way to prove that they visited community members. Community members occasionally came to the health center complaining that they had not been visited by a CHW. The CHWs said that their Geohealth records helped them prove visits to these community members.

### Main benefits of the Geohealth mobile application

The CHWs perceived a number of benefits from using the Geohealth mobile application (see Table 3 for illustrative quotes). Overall, CHWs believed that the mobile application helped make their work more efficient. They appreciated the Geohealth mobile application for the ease with which it let them access information from previous visits. The application helped them keep track of which families they needed to visit in a given month. Finally, the mobile application eliminated the amount of paper CHWs had to work with and lightened the load that they had to carry on a daily basis.

**Table 3:** Benefits of the Geohealth application

Saves time spent on bureaucratic paper work	Geohealth makes things faster, you can quickly make lists, filter community members, find all of your hypertensives, and find particular families. So I think Geohealth makes things faster, but the information is safer on paper. It is faster with Geohealth at the end of the month because we have one day of accounting, so to speak. We used to have to count manually—hypertensive by hypertensive— going through all the forms. Before we had to look through each folder and manually count, manually look for each kid under age 2. Geohealth is way better.
Faster access to information	Geohealth makes the quality of our work better. It helps me make lists of community members in my area. I can choose, for example, to see only the pregnant women in my area, or only the 2 year olds, and then I have a complete list right away Another example, let’s say a community member has a question about an appointment from a few months ago, like when it was. I can just pull it up right away! Before Geohealth, I’d have to go back to the health center and look through piles of paper to find that information. But it is all stored in Geohealth. Or another example, if a community member has a doubt about what medication she was prescribed. I can pull it up on Geohealth in her house during the visit. I don’t have to come back later. “It [Geohealth] is really important. It is fundamental. I write down all sorts of information during visits like diabetic’s insulin levels and blood pressure. I write it in the observation field. Then at their next visit I look up their values from last time to see how they’re doing. This is different from the paper system, because paper is saved in the health center. And then I wouldn’t have access.”
Weight	I like it because it means less paper for me. In our work we walk a lot, and our backpacks are heavy, and we end up taking off our backpacks in community members’ homes and forgetting them there. Biggest difference between Geohealth and paper? My bag is lighter with Geohealth! I don’t have to carry as much paper. I typed the family number and it had all the information about that family. It was the size of my hand versus [carrying] a mountain of paper. Better to have the device. If Geohealth had all the forms it would eliminate a ton of paper and work for us.

#### Saves time spent on paperwork

The main advantage of Geohealth was that it stored information that the CHWs regularly needed in one location and in an organized fashion. This saved significant time when CHWs were filling in required paperwork at the end of the month. Without Geohealth, they had to sort through community member charts to manually count the number of people in an area that had hypertension, diabetes, or other characteristics that represented health priorities as determined by the FHS. The Geohealth program produced a count of community members with these characteristics for CHWs and helped avoid errors when doing manual monthly counts.

#### Faster access to information

The CHWs used the program to access data from previous visits to answer community members’ questions, review information to plan home visits, and make monthly counts of health priority information. All of these tasks required sorting through paper charts before Geohealth.

#### Weight

Finally, Geohealth lightened the physical load that the CHWs carried during the day. The CHWs work long hours walking from house to house on their home visits. Many of the neighborhoods where the CHWs work are topographically arduous, and CHWs often had to climb several flights of stairs to reach community member residences. Thus, not having to carry reams of paper forms was perceived as a key benefit.

### Main barriers to effective use of the mobile application

Despite the advantages noted above, 76% of the CHWs reported that they did not use Geohealth regularly during home visits. They cited a variety of technical and social barriers to using the mobile application (see Table 4 for illustrative quotes). Both the mobile application itself and the smartphones they used created technical barriers to the use of the application during home visits. A majority of the CHWs felt that using paper was more practical than using the mobile application. Social barriers included community member’s perceptions of the mobile application and safety concerns about carrying around an expensive smartphone.

**Table 4:** Barriers to effective use of Geohealth application

Technical barrier: inefficiency	The negative side is that Geohealth would crash or freeze in the middle of a visit. It would stop all of a sudden and oh, how it would make you angry. You can get an interview done in less time than it takes to open the application. [The mobile application] wasn’t a good interface. You had to jump from screen to screen in a random order to get what you wanted. You could organize the pages so it is more efficient. You had to jump between all these different screens. It would be better to have it all on just one screen. Sometimes there is duplicated data [with the mobile application]. So I always write down my numbers and can check them against Geohealth. If one day Geohealth says I have 202 families, but I have written down that I have 198, and I haven’t registered any new families, I know there is a problem in Geohealth. Geohealth doesn’t always remember the changes we make. For example, when a community member dies, we delete the record from Geohealth. But then later it keeps popping up again, and we know that person is dead. We take community members out of the system and then they reappear. Paper doesn’t delete the information. The only annoying thing is when I type a huge amount about the visit, then look up from the phone to the community member, and accidentally hit the home button. The phone leaves Geohealth and I lose everything I wrote. I accidentally delete it all.
Technical barrier: signal strength	It’s just the device that doesn’t work. We always have to wait for the signal and for it to start working. The signal. The signal could be better. Sometimes it gets stuck searching for a signal and you have to force quit. Then you have to re-type everything. That’s annoying.
Technical barrier: the device	I used it [Geohealth] for just over a year, but then the device broke on me. I sent it in to be fixed. After 6 months, it came back, but it was still broken in the same way. Now I don’t have one [a smartphone]. The biggest problem is the reception. After that, the battery. After charging it in the morning, the battery is dead by the afternoon. The device is terrible but the computer system helps us a lot. I use it a lot on the computer. I didn’t like the Geohealth phone. The computer was much better. I couldn’t pay attention to the community members when I was using the phone. Cell phones are distracting. Their keys are too small. I update Geohealth information on my computer at home.
Social barrier: community member perceptions	The community members, especially the older ones, complain. They say, look at me! I’m telling you a story, pay attention! Eye contact is so important. Sometimes a community member doesn’t say he’s in pain, but you see it in their face. Keeping an elderly person company has no need for Geohealth. The idea of having Geohealth is to make your life easier, but that doesn’t mean it makes the lives of the community members easier. I use Geohealth on the computer. Like I said, I don’t use the Geohealth cell phone. It sits in the closet. I never use it. It isn’t good for community members. You have to listen to them, and you can’t listen if you are sitting there trying to type. Community members lose their connection with you when you’re on the cellphone. You can’t show them that you’re not texting and that you’re completing the work if they’re illiterate.
Social barrier: safety	I work in a risky area. There is a lot of drug trafficking. If the police pass by, and they see my typing on the Geohealth, they think I’m alerting drug traffickers that they are here. Or if a drug trafficker sees you typing they think you are alerting the police. But I also have to be more discrete with Geohealth. There is some danger of robberies. We are a little hesitant to use it on the street. We have to be. Even though it is an old phone, someone might think it is nice. Anything we use has to be discrete so it doesn’t catch the attention of thieves. What I do to prevent it from being stolen is I show the drug dealers the phone. I know them because they are the same kids from the preschool where I worked previously. I show them the phone and say ‘you could steal it if you wanted to. You’d be able to unlock all the functions that are blocked. But I’ll have to report it because it isn’t mine. It is public property, so I’ll have to report it to the police. I have no choice. And if I lose it, I have to pay for it out of my own salary.’

#### Technical barrier: inefficiency

Many CHWs reported finding the mobile application slow. Some noted that they did not like the interface because it was hard to move between screens to access needed fields. They reported that the app took a long time to load, froze frequently and shut down without warning. Thus, they concluded that they could write paper notes faster than using the application. The CHWs mentioned that data were frequently lost from the system when the application froze. One CHW was afraid that she would lose her data on the smartphone and others mentioned that sometimes data randomly disappeared from the system. The CHWs did not encounter these technical difficulties with the computer program.

#### Technical barrier: signal

Many CHWs mentioned that poor cellphone service was a barrier to using the mobile application. In theory, the mobile application does not require cellphone service to operate in the community. The application is supposed to save data locally until it makes a connection with the server and then send data on the phone back to the Geohealth database. CHWs indicated a variety of problems when the phone had no signal: the cellphone loaded slowly when there was no service; it froze as it searched for service; it did not allow them to save the data without service; or the application did not start without service.

#### Technical barrier: the device

The CHWs noted that the smartphone itself could be a barrier to their work. CHWs had different smartphone models depending on when they started using Geohealth. Their comments generally revolved around the keyboard size, battery life, and lack of access to someone to fix the phone if it was broken. One of the CHWs who used the mobile application during home visits said that she used it until the battery died and then switched to paper. Many preferred using the Geohealth computer program when they returned to the primary care center to using the mobile application during home visits. Using the computer had the advantages of a bigger keyboard and faster processing speed.

#### Social barrier: community member perceptions

Community member perceptions of the mobile application constituted one of the main social barriers preventing the CHWs from using it during home visits. CHWs generally did not think that community members perceived Geohealth positively. Using the application changed the nature of the home visit; it required that the CHW concentrate on typing and prevented them from maintaining eye contact with community members. Many community members, especially the elderly, felt that the application interfered with social interaction. They wanted the CHW’s full attention during home visits. Additionally, some of the CHWs found it hard to give community members their full attention if they were using the phone, and they noted the importance of eye contact when building trust with community members.

#### Social barrier: safety

Additionally, some CHWs mentioned safety concerns as a barrier to using the phone. They worried about their personal safety when seen with the phone; fear of the phone being stolen; and fear of the cellphone getting damaged or broken. The CHWs reported that they conducted many home visits in the street and that they were uncomfortable using the phone in these circumstances.

### Recommended improvements to Geohealth

The CHWs identified key areas where the efficiency and usefulness of the mobile application could be improved (see Table 5). The most common suggestions were including all of the forms CHWs use in Geohealth and including a communication feature. Other logistical suggestions included a calendar function, electronic signatures, health education information, and an alert system in the mobile application. Individual CHWs had innovative ideas for the app that are described in more detail below. Finally, the CHWs often made suggestions about improving the hardware that was used to run Geohealth.

**Table 5:** Recommended improvements to Geohealth

Include more data	Include ALL of our paperwork. Have you seen our paperwork? [she goes into another room and brings back a stack of different papers that CHWs use for their work and for their monthly tallies of data at the end of each month. She shows me each form and how tedious it is to do the counts.] All of this could be done automatically on Geohealth. And then at the end of the month, we’d just print it and be all set. The end of the month days when we have to do these counts. It’s very tense. It is a lot of work: everybody is stressed out. Sometimes I wake up at 3 am to finish. And when you’re new, it is even worse. Oh, it’s a real headache. The point of technology should be to get rid of all these papers and little papers. They should integrate ALL of our forms, not just form A and the home visits.
Communication	It would be wonderful if we could send and receive messages because right now we use WhatsApp. We could contact the family directly if we needed some kind of information. If community members could send text messages with questions for the CHW’s from their personal phone to the CHW’s Geohealth app, that would be incredibly helpful. It [the mobile application] couple be improved if it allowed more communication between the team. I have no way of communicating with my team except for my personal cell phone. I don’t give my WhatsApp number to my community members because I need a barrier between personal and work life—it doesn’t work out to mix them. I have to cut it off somewhere. It would be nice if we could send texts with the Geohealth phone. We could alert community members if their exam results arrived, or if they have an appointment coming up. Something like WhatsApp to communicate with community members. My Geohealth phone sits in the closet. But if I were able to use it to send messages to community members, then I would use it.
Logistic improvements	Also, there is no way to get a signature on the cell phone. I have all my community members sign on paper when I visit their house so that I have a way of keeping track, or proving that I visited them, so they can’t say I didn’t go later. I ask for a signature at every visit. Why don’t they add a function for a digital signature? Like a fingerprint? They could just put their thumbprint into the cell phone to sign. That also helps the illiterate community members too. It would also be nice if it had little alerts or reminders for individual community members who needed exams. Like if I went to someone’s house, when I opened the visit form it would remind me that this person needs a mammogram, and I could talk about that at the visit. I don’t always remember everything every person needs. This would make it more effective. She said that the information collected could be used to educate community members. It could be used to show them “research” about their health. She gave the example of community members who buy medication on the street and ask her about it. Again, she would like to be able to give them information that she doesn’t know during the visit rather than waiting a month to get back to them. She would also like to record her visits. She also thought that Geohealth could be used to connect community members to health resources. For example, it could have our work cards—instead of writing down when we come and leave work each day, it could be recorded in Geohealth. It could have our teams, calendars, and keep track of all the work I do. I know what I do each day so I’m not keeping track for myself. It would be nice if there was a built-in tracker of how many visits we had done each month to keep track of our monthly goals. Everything could be compact in this one device. It is necessary for us to have more information about health. Most of what I learn about health is from the news—TV journals. Sometimes our community members see health programs on TV and it is something we don’t know. We say, oh, really? I would want that information in my smart phone.
Individually suggested innovations	There are Facebook groups for lots of things. I had a cousin who had her gallbladder removed and needed advice. She found a Facebook group for other patients recovering from the same surgery. That would be good. We have a garden here that we use to teach community members and CHAs about herbs and teas for different conditions- like hypertension. You could have different groups with the garden. There are many people who like gardening, especially old people, who don’t have good places at their house. Some people don’t like gardening they just like drinking tea. You could have different groups for those who like gardening and those who like drinking tea. I think you would get more people involved that way, off their couch and away from the tv. We have a problem here. Old people think that when you retire you stop being active. It’s hard to change their thinking. It would be good to provide activities for them and I think more would come if you could remind them. Geohealth could be programmed to help us understand the demands of the health center. How many visits, how many medications…it would make predictions based on the data. We could type in how much insulin patients are using, and if they are getting it but not using it at home. We could also use it to motivate community members by showing them how they are improving. For example, if a community member was taking good care of a wound but didn’t think it was healing, we could show them a photo from last week and say “look here! It is better.” It is a way of incentivizing community members to take care of their wound because we are documenting it. I’d use a camera to take photos—to motivate community members. For example, we have groups of hypertensives, diabetics, and we do activities with them. We have workshops and things. It is important to take pictures of these events. To document our work, and it is a way of motivating them to come. People love photos, the community members love photos. We observe the entire house. We’d like to take a photo, sometimes we use our own phone to take photos. I take pictures of trash pile. Sometimes I put pictures of trash on social networks to show the government how things are here. I take pictures of things that call my attention.
Improved device	How can the system be improved? A tablet with a stylus would be great, because they we could write with the stylus... it is more natural than typing. I think the community members would be more comfortable with that too because it doesn’t look like we are typing on WhatsApp. It is also faster than typing. Tablets would be better perceived by community members because the community member could see what I am doing on the screen and the keyboard would be bigger. I would only change the device - it deletes the visit. Also, having more computers to enter the visits who help. We have to make a summary for the nurse and this is a lot of work. It takes a lot of time to enter all of the visits in the computer.

#### Incorporate all paperwork into mobile application

Most notably, the CHWs wanted Geohealth to include all of the required documentation that they collected. Geohealth only included templates for recording one of several forms that they were required to fill out monthly. The CHWs believed the application could also facilitate their other required paperwork, such as data collection on chronic health conditions. Additionally, the CHWs keep track of vaccinations and screening tests recommended for each community member. Yet, Geohealth did not have features to record and keep track of these. The CHWs believed the application could also facilitate recording and monitoring of other key health and screening information for their assigned community members.

#### Communication

Another commonly mentioned improvement was the ability to use the mobile application to communicate with other CHWs, their health center team, and community members. Many CHWs mentioned that their teams already used an application called WhatsApp to communicate with each other. However, the CHWs had to pay to use this application (WhatsApp became a free application after the conclusion of this study). Some of them did not want to use personal phones to contact community members in order to protect their privacy. Additionally, the CHWs had no way to communicate confidentially with community members in between home visits while also avoiding using personal phones. For example, the CHWs wanted to use Geohealth to give community members quick feedback on their questions for the doctors and nurses at the health center. The CHWs noted that a function to facilitate communication in the mobile application would save the CHWs time and strengthen their ability to aid community members.

#### Logistic improvements

The CHWs suggested a few other improvements to the mobile application. These included functions that enabled them to schedule appointments at the health center, send reminders or alerts, store photos, access personal and health center calendars, as well as connect to educational materials in Geohealth. Additionally, the CHWs wanted to store electronic signatures in the database. Some CHWs recommended improvements to the user-interface such as new data fields.

#### Individually suggested innovations

Individual CHWs mentioned unique innovations for the Geohealth mobile application or other applications that could aid their work. One CHW mentioned that she would like to see Geohealth used as a social networking platform to connect community members based on their health needs or hobbies such as gardening. Another wanted to use Geohealth to help community members organize their medications. One CHW wanted to add geographic points of interest relevant to community health such as open trash piles to the database. A final suggestion came from a CHW who thought the application could be used to perform a needs assessment for the health center. She wanted to use it to determine how many staff members the health center needed as well as what medications they should stock.

#### Improved device

Several CHWs mentioned that better equipment would enhance the function of the mobile application. Some mentioned that a tablet or a device with a bigger screen and keyboard would be helpful and mitigate the technical barriers of trying to enter and review data on a small smartphone. The benefits of a tablet would be that they could use it for community member education, to show community members what they were doing while they entered data in Geohealth, and to share data with the community members. Additionally, they thought a tablet would look more official and professional. Other CHWs simply mentioned that a higher quality smartphone might freeze or shut down less than the current devices.

## Discussion

The perceptions of these CHWS from six primary care centers in São Paulo, Brazil, of the benefits of and barriers to using the Geohealth mobile application provide important lessons for m-health tools designed for CHWs. While almost all CHWs found the overall Geohealth computer program useful to help compile and organize data, many had mixed experiences and views of the mobile health application. They reported several benefits of the mobile application such as saving time with paperwork, organizing the data that they needed to collect, and by replacing sheaves of paper, reducing the weight that they carried in the field. However, there were many technical and social barriers to the successful adoption of the m-health tool. Key among these were poor quality hardware, faulty software programs, perceived safety in the field, and negative community member perceptions of the program.

The Geohealth computer program helped the CHWs efficiently complete a number of their required tasks such as gathering data on families served and generating counts of priority disease conditions of families served (e.g., diabetes). Geohealth recorded family home visits completed each month. However, few CHWs used the mobile application in home visits as had been the intention of program developers, with over three fourths of interviewed CHWs reporting using paper notes in the visits and only later entering the data on the mobile application or desktop computers at health centers. For these CHWs, the m-health tool was hardly saving time as they ended up recording the data twice.

Some technical barriers were intrinsic to the design of the application and could be addressed through re-designing features. CHWs appropriately shifted to other modes of recording data in the face of poor reliability of the application in the field and to avoid losing critical data. m-health tools will not completely replace use of paper and pen until CHWs can be confident in the reliability of the devices and networks. Overcoming some technical barriers may require better development of infrastructure, as cellphone coverage in some of the neighborhoods was spotty. Fortunately, Brazil has launched a new satellite (Satélite Geoestacionário de Defesa e Comunicações Estratégicas - SGDC), coordinated by the Ministry of Defense. This satellite should offer broadband internet to the whole country, including remote and difficult to reach areas starting in September 2017 such as those served by the CHWs in this study. Other barriers, such as difficulty typing on the small smartphone keyboards, could be addressed with additional training such as educating CHWs to use the already-present voice command functions that only one CHW reported successfully using. Tablets are currently being used instead of smartphones in a new project in this region as a result of the CHW’s suggestions. This has fixed the keyboard issues. Unfortunately, the concerns about theft and safety when carrying around expensive technology remain.

Social barriers to use were also perceived as significant. In the same way that patients complain about doctors focusing more on the electronic health records than on them, CHWs reported that community members complained when they focused on typing on their smartphones rather than conversing directly with them during home visits. As the current design relies on typing all answers, improved design (e.g., effective use of drop-down boxes) might help mitigate this barrier. Moreover, designing the m-health tool to better facilitate recording and following up on information gathered in discussions with community members could enhance the effectiveness of their home visits. Better design could enable them to follow up on health concerns mentioned in home visits and relay information from their home visits to other health care team professionals.

Our findings reaffirm the importance of effective development and iterative testing of m-health tools. The use of approaches such as user-centered design (UCD) is critically important. UCD helps to ensure that programs are easy to use and meet the needs of users [23, 24]. UCD involves end-users throughout the development process so that technology supports tasks, is easy to operate, and is of value to users [24]. Using a well-designed UCD process is necessary to help ensure that tools are culturally appropriate, beneficial to the users, and easy to use and understand [25].

Our findings also point to additional features that would enhance the productiveness of CHW encounters with community members in home visits and communication outside of face-to-face visits. As several CHWs recommended, better patient-facing features could help CHWs identify and record community members’ current health needs, monitor progress toward meeting these needs, and provide more effective follow-up through text messages [26]. There is a growing body of evidence demonstrating the effectiveness of between-clinic visit supportive, educational, and reminder text messages [27–31]. Such messages could be generated through a mobile application or link to applications that CHWs and community members currently use (such as WhatsApp). Again, any additional features would need to be designed, developed, and implemented with significant input from CHWs and other key stakeholders.

Our study has a number of limitations. Most significant, we only interviewed a sample of CHWs from primary care centers in one region of Brazil who used one specific m-health application. Thus, their experiences and the issues they faced may not generalize to other health workers in other regions using other types of m-health tools. The barriers to use they reported, however, can inform other efforts to design and implement effective programs. Better software, newer devices, and slow-but-eventual community member acceptance of the technology are improvements that can reasonably be targeted and achieved**.** Future studies may include survey data to quantify different experiences with Geohealth and analyze the association between CHW characteristics and specific views of Geohealth.

## Conclusion

This study reinforces the potential of m-health tools to facilitate the outreach work of CHWS in low- and middle-income countries. However, such tools must be designed and implemented thoughtfully. Technical barriers related to both hardware and software as well as potential social barriers must be anticipated and addressed to maximize their efficiency and successful adoption. CHW input on the design of the tool should be sought to maximize its utility and minimize barriers to use.

## Abbreviations

CHW: Community health worker
FHS: Family Health System
LMIC: Low- and middle-income country
UCD: User-centered design
